# Effects of acute high‐altitude exposure on working memory: A functional near‐infrared spectroscopy study

**DOI:** 10.1002/brb3.2776

**Published:** 2022-11-02

**Authors:** Li Wang, Linqiong Sang, Yu Cui, Pengyue Li, Liang Qiao, Qiannan Wang, Wenqi Zhao, Qiu Hu, Najing Zhang, Ye Zhang, Mingguo Qiu, Jian Chen

**Affiliations:** ^1^ Key Laboratory of Extreme Environmental Medicine, Ministry of Education of China Army Medical University Chongqing China; ^2^ Department of Medical Imaging, College of Biomedical Engineering Army Medical University Chongqing China; ^3^ Department of High Altitude Physiology and Pathology, College of High Altitude Military Medicine Army Medical University Chongqing China; ^4^ Institute of Medicine and Equipment for High Altitude Region, College of High Altitude Military Medicine Army Medical University Chongqing China

**Keywords:** dorsolateral prefrontal cortex hemodynamic response, fNIRS, high altitude, working memory

## Abstract

**Introduction:**

Inadequate oxygen availability may lead to impairment of neurocognitive functions. The aim of the present study was to investigate the effect of acute high‐altitude exposure on the cerebral hemodynamic response and working memory.

**Methods:**

The same subjects performed working memory exercises with forward and backward digit span tasks both under normal oxygen conditions and in large simulated hypobaric hypoxia chambers, and a series of physiological parameters were evaluated. Functional near‐infrared spectroscopy was used to measure cerebral blood flow changes in the dorsolateral prefrontal cortex (DLPFC) during the tasks.

**Results:**

Compared with normoxic conditions, under hypoxic conditions, the heart rate and blood pressure increased, blood oxygen saturation decreased significantly, and the forward task had similar accuracy and response time, while the backward task had lower accuracy and longer response time. Neuroimaging analysis showed increased activation in the DLPFC during the forward task and deactivation during the backward task under hypobaric hypoxia conditions.

**Conclusion:**

Acute high‐altitude exposure leads to physiological adaptations. The abnormal hemodynamic responses of the DLPFC to hypoxia at low pressure reveal the disruption of neurocognitive function by acute high‐altitude exposure, which compromises complex cognitive functions, and provides a promising application for functional near infrared spectroscopy in the exploration of neural mechanisms in the brain during high‐altitude exposure.

## Graphical abstract

The current study adopted an experimental paradigm of digital span to evaluate acute high‐altitude exposure impairment on working memory. The subjects showed obvious physiological adaptation mechanism at acute altitude. The results of hemodynamic response confirmed the important role of left DLPFC in the process of working memory, revealed abnormal hemodynamic response of DLPFC caused by acute hypoxia exposure.

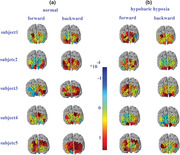

## 1 INTRODUCTION

Low‐pressure and low‐oxygen conditions have great impacts on people at high altitudes. It is well known that acute exposure to high altitude for several hours can cause significant changes in the physical and psychological parameters of the body, and in severe cases, cognitive function can also be impaired (Mc Morris et al., 2017; Terraneo, 2017). In recent years, a variety of neuroimaging techniques have been used to explore the neural mechanism of cognitive impairment in the high‐altitude hypoxic environment (Heinrich et al., 2019; Turner et al., 2015), such as prolonged reaction time and increased rate of judgment error.

Working memory is an important cognitive ability that consists of several phases, such as encoding, maintaining and retrieving given information. It involves the brain's temporary storage capacity and manipulation of information for performing complex cognitive tasks. The classical model of working memory is the multicomponent model proposed by Baddeley (2010), which divides it into four subsystems: the central executive, phonological loop, visuospatial sketchpad, and episodic buffer systems. The central executive system is an attentional control system. The phonological loop and the visuospatial sketch pad systems are responsible for the storage and rehearsal of verbal and visuospatial information, respectively. The visuospatial sketchpad system is devoted to the processing of visual and spatial information.

Based on the multicomponent model, the forward span is a short‐term memory task that is related to the capacity of the visuospatial sketchpad system, and requires storage and retrieval. Backward span would be more complex in cognitive terms, and is related to the execution process, the need to store information, and to manipulate the order of presentation to retrieve it accordingly. Many studies have reported that high‐altitude exposure leads to changes in working memory (Baddeley, 2010; Yan, 2014). During high‐altitude exposure, patients showed reduced attention and alertness and impaired executive function and memory. In addition, accuracy was lower in verbal‐working memory tasks, and the response time was longer in spatial‐and verbal‐working memory tasks (Foster et al., 2015; Li et al., 2010; McGuire et al., 2014; Wang et al., 2012). Although previous studies have reported changes in cognitive abilities associated with acute ascent to higher altitude, these conclusions have been predominantly obtained from observations associated with psychological scale analysis. In contrast, there are few studies on the changes in cerebral blood oxygen levels associated with acute exposure to high‐altitude hypoxia.

Recently, a noninvasive probing technology, functional near infrared spectroscopy (fNIRS), has been increasingly used for brain‐activity monitoring (Abtahi et al., 2020; Hu et al., 2019; Pinti et al., 2018). fNIRS uses two or more different wavelengths of light to measure levels of oxygenated and deoxygenated hemoglobin in the underlying brain tissue while test subjects perform various tasks, and provides independent measurements of multiple chromophores and gives a high temporal resolution. Because it is relatively an anti‐motion artifact, portable and flexible, it has been widely used in the study of visual, auditory, motor and cognitive stimulation, as well as in the study of children, adult patients and other atypical populations. Many studies have shown that fNIRS technology has good spatial and temporal correlation and is particularly suitable for studying the state of blood flow and function in the prefrontal cortex.

The prefrontal cortex is an important region for maintaining cognitive function, and previous studies have provided neuroimaging evidence that prefrontal cortex activation is associated with reactive working memory. Left prefrontal cortex activation is related to working memory in adults and children. Obvious hemodynamic responses changed in the left prefrontal cortex during visual working memory tasks, and prefrontal cortex activation was associated with both maintenance and attentional monitoring processes during visual memory tasks (Basten et al., 2011; Emch et al., 2019; Marshall et al., 2015). In particular, the dorsolateral prefrontal cortex (DLPFC) is considered a key region involved in the processing of key information in working memory. Bilateral DLPFC activation increased with the increase in memory workload, which may reflect the recruitment of additional cognitive resources to maintain the performance of complex tasks.

The study intended to apply fNIRS system to provide a more flexible solution to measure the performance of working memory tasks for subjects with different natural conditions. Compared with fMRI, the fNIRS system is more favorable for obtaining brain hemodynamic responses under real‐life environmental conditions. To study the impacts of an acute altitude environment on human cognitive function in a short period of time, for example, taking an airplane to the plateau, we used two of the digit span tasks (forward and backward) to examine working memory performance in healthy people under normoxic and hypobaric hypoxic conditions. Two hours after entering the simulated low‐pressure anoxic chamber plateau, we estimated changes in the blood oxygen concentration of the DLPFC, and explored the effects of acute altitude exposure on working memory. We aimed to explore (1) whether the hemodynamic response of the DLFC is influenced by acute hypoxia exposure and (2) whether acute high‐altitude exposure impairs working memory.

## 2 METHODS

### 2.1 Participants

Data were collected from 11 healthy adults aged 21–23 at the Army Medical University site. All participants provided informed consent approved by the Army Medical University Ethics Committee. Only two subjects completed the experiment in normoxic conditions but did not complete the experiment in the hypobaric hypoxia conditions, and four subjects performed poorly due to hypobaric hypoxia. The data from 5 young adults (ages 21–22, mean = 21.6 ± 0.3, two females) were entered into the final analysis.

### 2.2 Instrument

**FIGURE 1:**
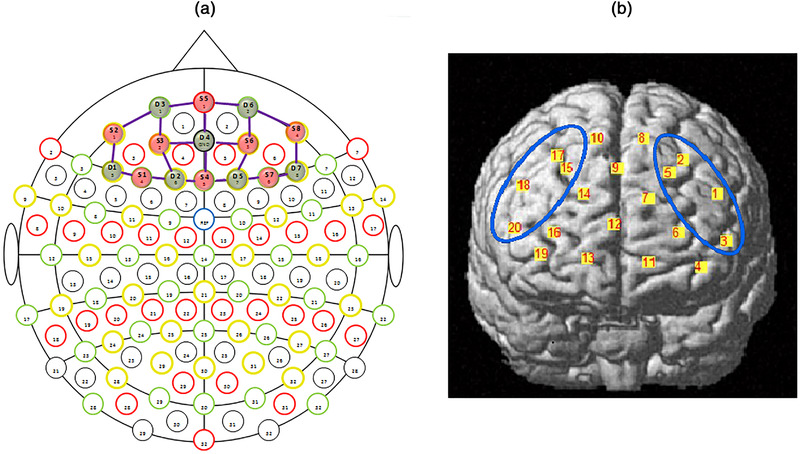
Configuration and cortical position of the fNIRS probe: (a) configuration of the fNIRS probe. Red circles represent light sources, blue squares represent detectors, and green lines represent the nearest source‐detector pairs (channels) to measure the brain activities. (b) Coregistered positions of the optodes on a standard brain atlas. The anatomical position of each channel on the brain atlas is reported in detail in Table 1. The channel involved in DLPCF: channels 1, 2, 3, and 5 are the left DLPFC and channels 15, 17, 18, and 20 are the right DLPFC

A continuous wave dual‐channel dual‐wavelength (760 nm, 850 nm) near‐infrared spectroscopy (fNIRS) system was used to obtain the hemodynamic activity of the prefrontal cortex in each subject during the task. We used the NIRScout system (NIRxMedical Technologies, LLC. Los Angeles, USA) to record fNIRS data. The system consisted of 7 LED emitters and 8 detectors, covering the prefrontal cortex. There were 20 channels, with an average distance of 3 cm from the source and detector. The sampling frequency was 3.91 Hz. The channel positions and corresponding cortical areas are shown in Figure 1. The three‐dimensional (3D) digitizer (patriot, Polhemus, Colchester, Vermont, USA) was used to measure the three‐dimensional coordinates of the optodes. NIRS_ SPM software was used to estimate the optical path, channel and MNI coordinates of each channel.

### 2.3 Experimental procedure

The same subjects performed working memory experiments both in the normal and large hypobaric hypoxia chamber model conditions. The simulation of high‐altitude conditions was carried out in a large low‐pressure chamber with an atmospheric pressure of 462 mmHg, which was used to simulate an environment at an altitude of 4000 m. The effect on the body is equivalent to inhaling 12.8% of the oxygen concentration in the plain. The subjects completed the experiment under normoxic conditions and then entered a simulated hypobaric hypoxia chamber 4 h later. After 45 min of slow depressurization, the preset simulated altitude (4000 m) lasted for 2 h, and the experiment was carried out in a hypobaric hypoxic environment. In a series of experiments, we evaluated a range of physiological parameters in both the normal and large hypobaric hypoxia chamber models, such as heart rate (HR), systolic blood pressure (SBP), diastolic blood pressure (DBP), and blood oxygen saturation (SaO_2_).

Working memory was measured using a forward (positive order) and a back digit (reverse order) span task (Tian et al., 2014). The backward task was more difficult than the forward task. The procedure consisted of 12 blocks, 6 of which were forward blocks and 6 were backward blocks. At the beginning of every block, a black symbol + symbol cue was presented for 1000 ms. Subsequently, the task was presented. Six numbers from 0 to 9 randomly appeared on the screen, and each number remained on the screen for 1000 ms, which was the encoding phase of the task. This was followed by a blank screen (10 s), which was the maintenance phase of the task. Finally, a series of numbers appeared on the screen, and the subjects had to complete sorting within 10 s by clicking the number button on the screen (positive order/reverse order), which was the retrieval phase of the task. A 10 s rest period was given before the next block. The memory task included three phases: encoding, maintenance and retrieval. To ensure the reliability of the experimental data and exclude accidental factors during training, the forward and backward tasks together required more than 30 min of training so that the accuracy rate of the subjects was truly over 95%.

### 2.4 fNIRS data preprocessing

The fNIRS data were preprocessed using the nirsLAB analysis package (v2018.05, NIRx Medical Technologies, LLC. Los Angeles, USA). Unacceptably high channel noise levels (CV threshold < 25%) were removed, contaminated data were replaced by linear interpolation by semiautomated smoothing, data were subjected to bandpass filtering (0.01−0.2 Hz), and intensity was converted to changes in optical density and then to changes in the relative concentration of oxyhemoglobin.

### 2.5 Statistical analysis

The fNIRS data (oxyhemoglobin [HbO] and deoxygenation [HbR]) over all channels were averaged, calculating the mean value of each subject and condition. This study compared average oxyhemoglobin concentration changes of hemodynamic responses in the DLPFC between baseline and task levels for each participant. The baseline was defined as the average concentration during the 10‐s period before each experiment. Next, the general linear model (GLM) was used to perform statistical analyses to quantify the amplitude of the hemodynamic response and detect significant cortical activation using a least squares estimated procedure. The design matrix included a boxcar regressor (stimulation condition) convoluted with the typical hemodynamic response function (HRF) provided in the nirsLAB analysis package, and smoothed with a 4 mm full width Gaussian kernel at the half maximum. The time series of each channel was high pass filtered at 1/128 Hz to eliminate low‐frequency interference. *T* statistic analysis was performed for a single subject to calculate the hemodynamic responses of each channel, which were then FDR corrected. A *p* < .05 was considered statistically significant. Finally, the activation maps induced by task stimuli were calculated and projected to the cortical surface.

The physiological and behavioral data were analyzed by SPSS‐22 software using descriptive statistics (mean score and SD) and paired *t*‐tests. A paired *t*‐test was used to compare physiological and behavioral data from normal and hypobaric hypoxia conditions.

### 2.6 Positioning of optodes and electrodes

**FIGURE 2:**
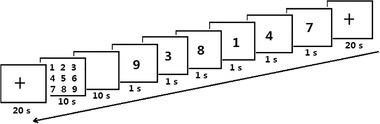
Experimental of digital span. A black symbol “+” reminder the task to begin immediately for 1000 ms. Six numbers from zero to nine are then randomly displayed on the screen for 1000 milliseconds each, followed by a blank screen for 10 s; finally, a series of numbers appeared on the screen, and the subjects were asked to complete the sorting within 10 s (in positive/reverse order), rest 20 s before next task. The backward task (reverse order) is more difficult than forward task (positive order)

The neuroanatomical position of each channel was located by a three‐dimensional Patriot Digitizer (Polhemus, USA). We selected five points as the reference points: Nz (nasion), Iz (inion), AL (left preauricular point), AR (right preauricular point), and Cz (central zero). The recording pen recorded the positions of the five reference points and then recorded the positions of all transmitting and receiving optodes in turn. NIRS_SPM (http://www.nitrc.org/projects/nirs_spm/) software was used to obtain the spatial coordinates of the human brain. Individual location data in the direction of the channel were registered separately to the standard Montreal Neurological Institute (MNI) space. We took the average coordinate point of each channel across all subjects as the position of each channel. The anatomical position of each measurement channel is shown in Figure 2. The areas covered by the fNIRS probes were mainly the DLPFC (Brodmann areas 9 and 46). The left DLPFC cortex was covered by channels 1, 2, 3, and 5, and the right DLPFC cortex was covered by channels 15, 17, 18, and 20 (Figure 2b, c and Table 1).

**TABLE 1:** Brain region distribution of fNIRS measurement channel after calibration by 3d locator

channel	Brodmann area	Anatomical area	Coverage percentage
CH01	9, 46	Dorsolateral prefrontal cortex	87.29%
CH02	9	Dorsolateral prefrontal cortex	64.86%
CH03	46	Dorsolateral prefrontal cortex	64.61%
CH04	10	Frontopolar area	86.59%
CH05	9	Dorsolateral prefrontal cortex	87.89%
CH06	10	Frontopolar area	100%
CH07	10	Frontopolar area	90.17%
CH08	8	Includes frontal eye fields	100%
CH09	9	Dorsolateral prefrontal cortex	69.82%
CH10	8	Includes frontal eye fields,	96.93%
CH11	10	Frontopolar area	82.20%
CH2	10	Frontopolar area	100%
CH13	10	Frontopolar area	93.13%
CH14	10	Frontopolar area	83.09%
CH15	9	Dorsolateral prefrontal cortex	73.01%
CH16	10	Frontopolar area	100%
CH17	8	Includes frontal eye fields,	87.28%
CH18	9, 46	Dorsolateral prefrontal cortex	100%
CH19	10	Frontopolar area	100%
CH20	46	Dorsolateral prefrontal cortex	68.38%

## 3 RESULTS

### 3.1 Physiological and behavioral tests

The physiological parameters are shown in Table 2. Compared with the normal environment (SBP: 114.00 ± 8.09; DBP: 71.20 ± 6.76; HR: 71.20 ± 6.76; SaO_2_: 96.6 ± 1.52), the systolic blood pressure, diastolic blood pressure, and heart resting rates of the subjects increased in the altitude environment, but oxygen saturation decreased (SBP: 121.60 ± 3.65; DBP: 67.00 ± 7.62; HR: 78.00 ± 9.57; SaO_2_: 84.4 ± 2.30). The paired *t*‐test showed a significant difference in SBP (*p* = .049) and SaO_2_ (*p* < .001) between normal and hypobaric hypoxia conditions. However, there was no significant difference in either the heart rate (*p* = .095) or the diastolic blood pressure (*p* = .099).

**TABLE 2:** Physiological parameters of blood pressure, heart rate, blood pressure saturation

	Normal				Hypobaric hypoxic			
Physiological parameter	HR (b/min)	SBP (mmHg)	DBP (mmHg)	SaO_2_ (%)	HR (b/min)	SBP (mmHg)	DBP (mmHg)	SaO_2_ (%)
Subject 1	72.00	124.00	64.00	97.00	72.00	128.00	75.00	84.00
Subject 2	65.00	104.00	63.00	97.00	75.00	119.00	75.00	82.00
Subject 3	75.00	119.00	58.00	97.00	74.00	120.00	65.00	83.00
Subject 4	64.00	108.00	61.00	98.00	74.00	121.00	61.00	88.00
Subject 5	80.00	115.00	60.00	94.00	95.00	120.00	59.00	85.00
Average **±** standard	71.20 ± 6.76	114.00 ± 8.09	71.20 ± 6.76	96.6 ± 1.52	78.00 ± 9.57	121.60 ± 3.65	67.00 ± 7.62	84.4 ± 2.30

HR, heart rate; SBP, systolic blood pressure; DBP, diastolic blood pressure; SaO_2_, blood oxygen saturation.

The behavioral performance for all subjects, including reaction time (RT) and accuracy (ACC) in the digital span task, is summarized in Table 3. Under normoxic conditions, there were no significant differences in average values between the forward task (RT: 8.62 ± 18.7; ACC: 90.2% ± 0.03%) and the backward task (RT: 8.78 ± 27.7; ACC: 88.6% ± 0.153%). The digital backward task took slightly longer and was slightly less accurate than the digital forward task, reflecting another physiological phenomenon of greater difficulty in the backward task. During hypobaric hypoxia conditions, the subjects’ performance in the forward digital task (RT: 8.72 ± 8.7; ACC: 89.8% ± 0.10%) was quite similar to the normal state, but the performance of the backward numerical task was poor (RT: 9.58 ± 3.7; ACC: 77.2% ± 0.59%). There was no significant difference. The paired *t*‐test showed a significant difference in ACC during the backward task (*p* = .033) between normal and hypobaric hypoxia conditions; however, there was no significant difference in ACC during the forward task (*p* = .789). The paired *t*‐test showed no significant difference in RT during the forward (*p* = .519) and backward tasks (*p* = .058).

**TABLE 3:** Task‐dependent performance accuracy and react time

	Normal				Hypobaric hypoxic			
	Forward		Backward		Forward		Backward	
	RT	ACC	RT	ACC	RT	ACC	RT	ACC
Subject 1	8.4	90%	8.7	85%	8.9	88%	9.5	75%
Subject 2	8.1	92%	8.4	89%	8.2	87%	9.6	65%
Subject 3	8.9	89%	9.5	88%	8.8	90%	9.3	79%
Subject 4	9.2	92%	9.1	95%	8.9	95%	9.7	83%
Subject 5	8.5	88%	8.2	86%	8.8	89%	9.8	84%
Average **±** standard	8.62 ± 0.19	90.20% ± 0.03%	8.78 ± 0.28	88.60% ± 0.15%	8.722 ± 0.09	89.80 ± 0.1%	9.58 ± 0.04	77.20% ± 0.60%

RT, reaction time; ACC, accuracy.

### 3.2 [HbO] and [HbR] concentration changes induced by experiments

**FIGURE 3:**
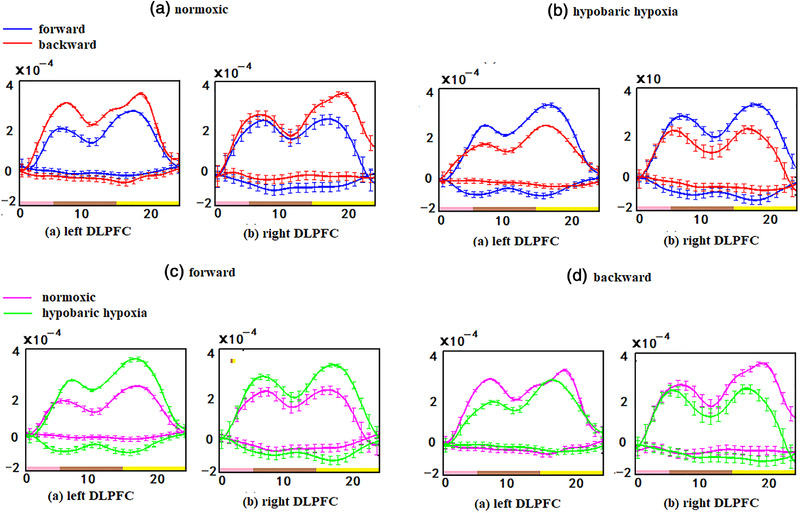
Mean task‐evoked [HbO] and[HbR] concentration of DLPFC in the group during both in the normxic and hypobaric hypoxia conditions: (A) mean [HbO] and[HbR] concentration on forward and back digital task of (a) left DLPFC and (b) right DLPFC during normoxic conditions; (B) mean [HbO] and[HbR] concentration on forward and back digital task of (a) left DLPFC and (b) right DLPFC during hypobaric hypoxia condition; (C) mean [HbO] and[HbR] concentration on forward digital task of (a) left DLPFC and (b) right DLPFC during both normoxic and hypobaric hypoxia conditions; (D) mean [HbO] and[HbR] concentration on back digital task of (a) left DLPFC and (b) right DLPFC during both normoxic and hypobaric hypoxia conditions. The pink line on the horizontal is encoding, the brown line is maintenance, the yellow line is retrieval, and the black line is rest

**FIGURE 4:**
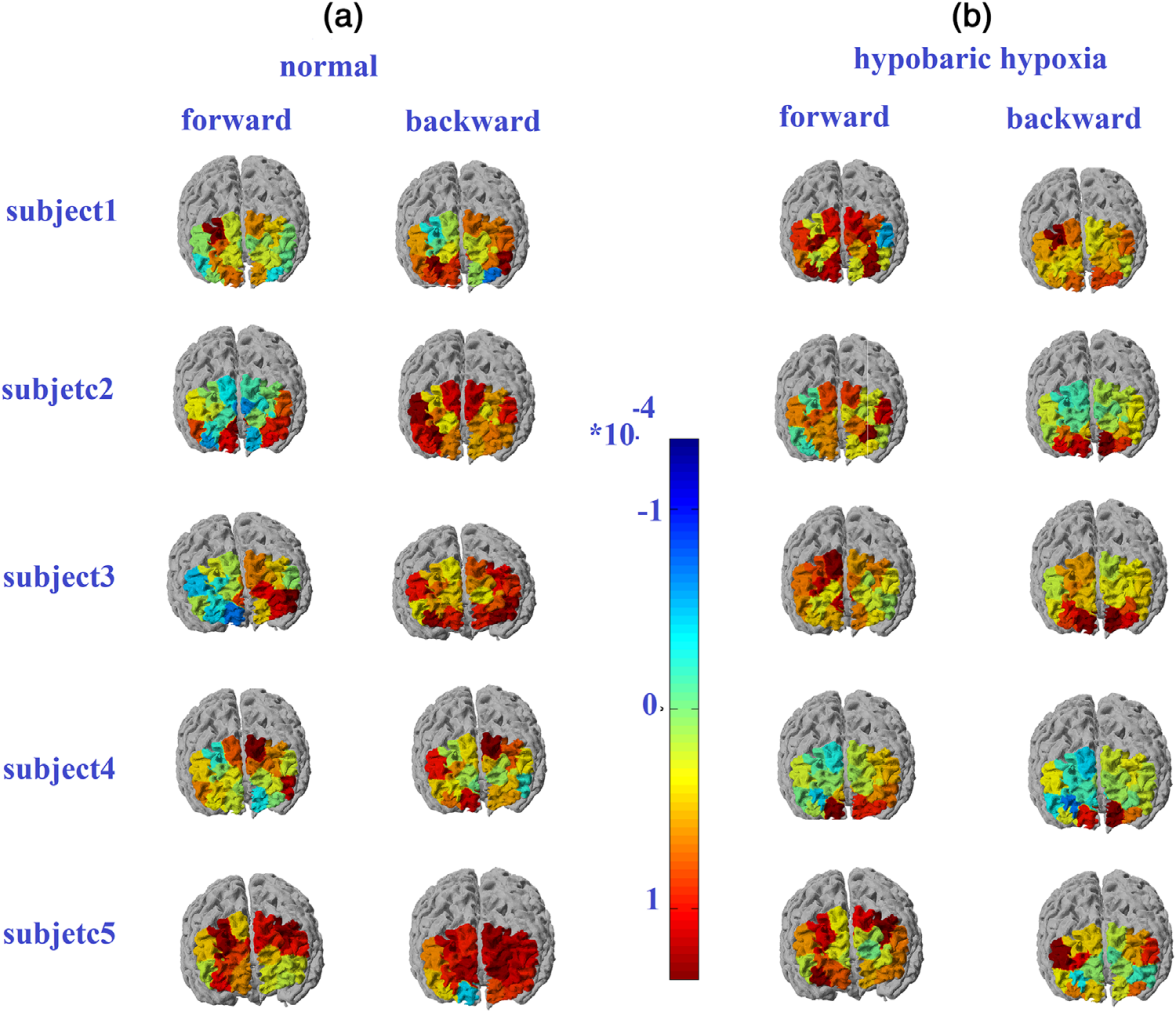
The images of the task‐evoked prefrontal activations in the each subject during both in the normal and hypobaric hypoxia conditions. The rows 1 through 5: brain regions activation evoked by the digit span task in each subject

The average [HbO] and [HbR] concentrations across 6 blocks for forward and backward tasks in the DLPFC across all subjects were calculated. As shown in Figure 3, in the current study, the channels 15, 17, 18, and 20 are the left DLPFC, and the channels 1, 2, 3, and 5 are the right DLPFC. The mean task‐evoked [HbO] and [HbR] concentrations in the DLPFC across all subjects in both normoxic and hypobaric hypoxic conditions are shown in Figure 4. In each period, the [HbO] signal began to rise slightly, peaking at approximately 7–8 s, then gradually decreased, bottoming out at approximately 15–18 s, then rising again to peak at approximately 20–22 s, and finally slowly fell back to the baseline in the remaining period. The [HbR] values followed a similar time‐course; however, the trend was inverted. Under normoxic conditions, the [HbO] concentration in the DLPFC on the backward task was higher than that on the forward task (Figure 3A); however, the results were reversed under hypobaric hypoxia conditions (Figure 3B). For the same forward task, the [HbO] concentration was higher under hypobaric hypoxia than under normoxia conditions (Figure 3C), but the opposite was true in the backward task (Figure 3D).

### 3.3 Task‐evoked brain region activation

The fNIRS data analysis showed activated cortical regions in each subject (Figure 4). Under normoxic conditions, the left DLPFC on the forward task and the bilateral DLPFC on the backward task were significantly activated in all subjects. During hypobaric hypoxia, the activated area was broader in most subjects (Subjects 1, 2, and 3) on the forward task; however, the activated area was smaller in most subjects (Subjects 1, 3, and 5) and almost absent (Subjects 2 and 4) in some on the backward task. Comparing DLPFC activity under the two conditions, it was found that the DLPFC BOLD response under the hypoxia condition was higher than that under the normal condition in the forward task, while some inactivation phenomena occurred in the backward task under the hypoxia condition.

## 4 DISCUSSION

This study aimed to identify the impacts of acute high‐altitude exposure on working memory. Working memory performance was measured using a digital span task combined with fNIRS recording of the BOLD response in the same subjects under both normoxic conditions and hypobaric hypoxia conditions. We found that (1) physiological adaptation to high altitude occurred, heart rate and blood pressure increased, and blood oxygen saturation decreased significantly; (2) under normal oxygen conditions, DLPFC activation in the left hemisphere was stronger in forward and backward tasks, confirming that the DLPFC in the left hemisphere plays an important role in working memory; (3) the accuracy of the backward task under hypoxia was significantly lower than that of the forward task under normal oxygen conditions. This indicated that acute high‐altitude exposure induced poor performance on complex tasks; and (4) the activation of bilateral DLPFCs increased in the forward task but not in the backward task under hypoxia, and the complex task may be more affected by acute exposure to high altitude.

### 4.1 Experiments during normoxic condition analysis

#### 4.1.1 Physiological and behavioral data

The normal physiological index of the human body is an important standard to measure health. It includes heart rate and blood pressure. In this study, all of the subjects' physiological indices were normal under normoxic conditions, indicating that they were in good health.

In this study, subjects responded faster on the forward task than on the backward task. In addition, under normal conditions, the accuracy of the forward task was slightly higher than that of the backward task. The difficulty of the memory task may increase with increasing memory load. Our results were consistent with the reduction in processing speed and working memory caused by the complexity of the task, as has been shown in a large amount of literature (Rovetti et al., 2021; Wells et al., 2018). According to the classic multicomponent model of working memory, there is no manipulation of information on the forward task, only storage and retrieval, so it is easier to complete; however, the backward task needs to store information and manipulate the order of presentation to retrieve it accordingly, so it is difficult to complete. At the behavioral level, these results supported the view that increased cognitive load will lead to deterioration of memory performance.

#### 4.1.2 Hemodynamic response

When the DLPFC was activated, activation was stronger in the left hemisphere during the forward task and much stronger in the left hemisphere during the backward task. An fMRI study showed that the right middle frontal gyrus was strongly activated in spatial memory tasks, while the left middle frontal gyrus was more strongly activated in non‐spatial working memory tasks (Brian Nils et al., 2005). Other fMRI studies have also found that the dominant hemisphere of spatial working memory was the right hemisphere, and activation was mainly in the right frontal lobe. In the nonspatial working memory condition, the dominant hemisphere was the left hemisphere, and the left frontal lobe was the most active (D'Esposito et al., 1998). In this study, we confirmed that the left DLPFC played an important role in information encoding and working memory retrieval, indicating that near infrared spectral imaging technology could accurately measure changes in HbO_2_ concentrations in cortical regions, thus predicting cortical neural activity and providing a promising technique for clinical diagnosis and research. The backward task produced a significantly higher HbO_2_ increase in the DLFC than the forward task. In addition, the brain response in the backward task was spatially broader than that in the forward task. These results were in accordance with previous fNIRS and fMRI studies that showed higher and broader activation during complex cognitive tasks, and this was also consistent with previous neuroimaging studies that revealed stronger and broader activation during complex cognitive tasks. Molteni et al. confirmed that increasing task difficulty resulted in increased HbO_2_ concentrations (Ayaz et al., 2012; Molteni et al., 2012). Molteni et al. (2004) also found higher brain activation in the prefrontal region during complex memory. Compared with simple tasks, these tasks were associated with stronger brain activation and broader involvement of brain regions.

### 4.2 Experiments during hypobaric hypoxia condition analysis

#### 4.2.1 Physiological and behavioral data

Acute altitude exposure leads to an emergency response of the body's mechanisms, such as increased heart rate and blood pressure. In this study, the subjects showed an obvious physiological response at plateau, with increased blood pressure and heart rate. In addition, blood oxygen saturation is also a sensitive indicator of hypoxia and its severity. Oxygen saturation dropped dramatically in all subjects. Blood oxygen saturation decreased, but heart rate increased significantly, which is a compensatory enhancement of the cardiovascular system to adapt to altitude. Our results confirm these physiological mechanisms at high altitudes.

Many studies have found that reaction times increase during acute exposure to altitude (Bahrke & Shukitt‐Hale, 1993; Chen et al., 2017; Ma et al., 2015). Previous reports showed that an increase in response times was a compensation mechanism that attempted to improve accuracy at the expense of speed (Bahrke & Shukitt‐Hale, 1993; Chen et al., 2017). In this study, repeated training ensures that the subjects' cognitive ability really meets the requirements and obtains reliable data. Consistent with previous studies, we found that acute elevation exposure reduced processing speed for the backward task, but there was no significant difference in response time for the forward task under normal and hypoxic conditions. This may be because the forward task was simple or not sensitive enough to measure the effects of altitude exposure, as previously reported, and the backward task was complex enough to measure the effects of increased response time due to altitude exposure.

#### 4.2.2 Hemodynamic response

Cerebral blood flow (CBF) is sensitive to changes in arterial blood gases, while neurovascular coupling (NVC) is the temporal link between neuronal metabolic activity and regional CBF, supporting adequate delivery of nutrients (Caldwell et al., 2018, 2017). When the subjects were rapidly exposed to high altitude, the cerebrovascular reactivity increased, and the cerebral oxygen saturation level in the local brain region gradually decreased with increasing altitude, indicating local brain anoxia of subjects at high altitude (Liu et al., 2018). Global cerebral oxygen delivery is maintained by increasing cerebral blood flow upon acute exposure to hypobaric hypoxia; thus, regional and localized changes in the hemodynamic response may explain neurocognitive impairment.

An abnormal hemodynamic response of the DLPFC has been reported as a potential marker of cognitive impairment in various neuropsychological disorders, but it has rarely been observed in previous high‐altitude exposure studies. Abnormal activation in the DLPFC, frontal gyrus, postcentral gyrus and other cortices was reported under acute and chronic hypoxia exposure. Pun et al. (2018) adopted cognitive assessment software and reported that sustained attention and selective attention were affected by acute high‐altitude exposure, and that precision tasks requiring long‐term focus may be affected and be more difficult to perform at high‐altitude exposure. Furthermore, Nation et al. (2016) performed a more precise neuropsychological test study. Acute exposure to a high‐altitude simulation resulted in rapid impairment of learning and memory. The main characteristics of memory dysfunction are memory coding and memory retrieval loss.

Our body can produce a series of reactions to adapt to the plateau when rapidly exposed to a high‐altitude environment. This is evident from the significant decreases in SaO_2_ and DBP and increases in SBP during acute exposure to hypoxia (Table 2). The strict training before the experiment enabled the activation of DLPFC to truly reflect the changes of cerebral blood oxygen during the cognitive experiment. We found abnormal activation after acute high‐altitude exposure in DLPFC brain regions; the hemoglobin concentration of the forward task increased (DLPFC activation) and that of the reverse task decreased (DLPFC inactivation). There were differences in the results for different hemoglobin concentrations, which may be related to differences in cognitive tasks. Physiological adaptation to hypoxia at altitude results in a higher hemodynamic status with a compensatory increase in hemoglobin levels for the forward task. However, acute high‐altitude exposure may have greater impacts on difficult cognitive tasks making them hard to perform well, which can be seen from behavioral data of backward tasks (Table 3). The hemodynamic status was low without a compensatory increase in hemoglobin levels in the backward task.

#### 4.2.3 Forward task

Many neurophysiologic and neural imaging studies have confirmed that the PFC is critical for many cognitive functions. Brain activation from subjects suggests that the HA group might have had very mild or even no deficiency in working memory, and that they might have utilized some adaptive strategies to ensure behavior performance. Yan reported that the BOLD response signal of the frontal cortex in the HA group did not decrease significantly, which is the landmark brain region for working memory; however, their previous study found that the gray matter volume of the frontal cortex in the HA group decreased (Yan et al., 2011). This suggests that the frontal cortex of the HA group was utilized to a greater extent, possibly by maintaining higher levels of attention during task performance.

Consistent with previous studies, we found that DLPFC activation increased in the forward task of Subjects 1, 2, 3, and 5 under hypoxia, which may suggest a compensatory mechanism of memory function when subjects suffer from neuronal loss due to hypoxia. The forward task requires subjects to sequentially display numbers from screen stimuli, and is related to the capacity of the visuospatial sketchpad. It is possible that subjects maintained the same capacity level of the visuospatial sketchpad to compensate for the decline in cognitive function caused by hypoxia. These results can be seen from the behavioral results; there was no significant difference in the response time and accuracy of the forward task for the subjects under normal and hypobaric hypoxia conditions. The increased activation in neuronal populations in the PFC needed to perform forward tasks further suggested the possible existence of compensatory mechanisms underlying the behavioral performance.

#### 4.2.4 Backward task

In extreme cases, exposure to high‐altitude conditions may be associated with decreased cognitive functioning of acute or even longer lasting duration. Yan (2014) applied a 2‐backward task exercise to study verbal working memory performance and corresponding neural activity in high‐altitude residents. They showed decreased activation in areas related to cognitive functions, such as the inferior frontal gyrus, middle occipital gyrus, and lingual gyrus. They showed longer response times and lower response accuracy in behavioral performance. Tian observed inactivation of the DLPFC in posttraumatic stress disorder, which may indicate active inhibition of DLPFC activity during working memory (Tian et al., 2014).

In this study, subjects did not show more DLPFC activation during the backward task under hypoxia but rather less activation. The backward task requires transformation to reorder the input digits in a reversed sequence after retention of the digits; this is considered to be part of the execution process. The central executive is an attentional control system that coordinates and schedules mental operations. It is difficult for subjects to pay attention to perform the backward task during hypobaric hypoxic conditions. This can also be seen from the behavioral results that the backward task is not very well completed. The backward task activation of the DLPFC was significantly reduced, and the response time was increased, reflecting the impairment of complex cognitive functions caused by acute altitude exposure.

## 5 CONCLUSION

The current study adopted an experimental paradigm of digital span to evaluate acute high‐altitude exposure impairment on working memory. The subjects showed obvious physiological adaptation mechanisms at acute altitude. The results of the hemodynamic response confirmed the important role of the left DLPFC in the process of working memory and revealed an abnormal hemodynamic response of the DLPFC caused by acute hypoxia exposure. Compared with normoxic conditions, the DLPFC exhibited stronger activation in forward tasks, which might be associated with possible compensatory mechanisms, thus increasing the blood supply to cope with acute hypoxia (Arleo & Gerstne, 2000; MacDonald et al., 2000). However, the DLPFC exhibited significant inactivation in the backward task, accompanied by a prolonged response time and a reduced correct rate, confirming the impairment of complex cognitive function by acute altitude exposure. This may be related to the frontalization hypothesis proposed by Duchaine et al. (2001). The human brain is a set of computational machines, each of which was designed by natural selection to solve adaptive problems. The human brain evolved and became more complex in the PFC. When the brain has less oxygen available, the tasks it can complete are simpler. According to the evolutionary analysis, the activation of the PFC in cognitive activities under extreme environments may be related to natural selection in the evolutionary process. The complex tasks are not easily activated on the PFC under hypoxic conditions because saving energy to survive is the most important.

### 5.1 Limitations

The current study had some limitations. First, the sample size was small, and there were five subjects in total, which may not be sufficient to assess the cognitive and neuroimaging changes caused by acute high‐altitude exposure. Second, we observed hemodynamic signals in the DLPFC during acute high‐altitude exposure and did not investigate the involvement of other cortical regions during working memory. For instance, the superior frontal gyrus, the middle frontal gyrus and the parietal gyrus may also be involved in cognitive function. Third, abnormal DLPFC activation was found to be due to altitude exposure, but the cause of the changes in brain networks remains unclear. fMRI studies have shown that functional and structural networks are disrupted in people during long high‐altitude exposure.

In the future, more studies should be conducted to expand the sample size for statistical analysis to obtain more reliable results. More probes should be used to cover more cortical areas and explore local activity and connectivity in a broader brain region to fully characterize the changes in hemodynamic responses caused by high‐altitude exposure to obtain more comprehensive results.

## CONFLICT OF INTEREST

The authors declare no conflict of interest.

### PEER REVIEW

The peer review history for this article is available at: https://publons.com/publon/10.1002/brb3.2776.

## Data Availability

The data generated and analyzed in this study are available from the corresponding author upon reasonable request.
